# Suicide mortality and associated factors among university students in Japan: A 5‐year nationwide epidemiological study (FY 2020–2024)

**DOI:** 10.1002/pcn5.70413

**Published:** 2026-09-21

**Authors:** Katsuhiro Yasumi, Takehiro Ibaraki, Yasuko Fuse‐Nagase, Toshiyuki Marutani, Hirokazu Tachikawa, Asumi Takahashi, Terumi Ishii, Toshinari Odawara, Chiaki Kawanishi

**Affiliations:** ^1^ Student Healthcare Center, Institute of Science Tokyo Tokyo Japan; ^2^ Center for Health and Wellness Ibaraki University Ibaraki Japan; ^3^ Department of Psychiatry Kinshicho Kubota Clinic Tokyo Japan; ^4^ Department of Medical Services Ibaraki Prefectural Medical Center of Psychiatry Ibaraki Japan; ^5^ School of Social Welfare Hokusei Gakuen University Sapporo Japan; ^6^ Health Support Center Waseda University Tokyo Japan; ^7^ Health Management Center Yokohama City University Yokohama Japan; ^8^ Department of Psychiatry Sapporo Medical University Graduate School of Medicine Sapporo Japan

**Keywords:** academic failure, COVID‐19 pandemic, suicide mortality, suicide prevention, university students

## Abstract

**Aim:**

Suicide is the leading cause of death among Japanese youth; however, nationwide data focusing specifically on university students remain limited. This study aimed to elucidate suicide mortality rates, sociodemographic characteristics, and presumed motives among university students in Japan during FY 2020–2024, following the onset of the COVID‐19 pandemic.

**Methods:**

Data were collected annually through the student affairs divisions of participating universities, covering a cumulative total of 13.5 million students, representing more than 90% of Japan's total university enrollment. We analyzed demographic characteristics, academic history, presumed motives, and campus health service utilization among identified suicide cases.

**Results:**

A total of 1526 suicide deaths or suspected suicides were identified. The male suicide rate (13.90 per 100,000) was significantly higher than the female rate (8.16), although the sex difference narrowed over the study period. Undergraduate and master's programs exhibited significantly higher suicide mortality rates compared with doctoral programs. Suicide deaths peaked in September and March, aligning with critical transitions in the Japanese academic calendar. The most common presumed motive was academic failure, followed by career concerns. Approximately 19% of cases involved a history of leave of absence or grade retention, whereas only 13.6% had prior contact with campus health services.

**Conclusion:**

Although suicide rates remained higher among men, the sex difference is narrowing. Academic failure and career concerns were prominent risk factors, and the low utilization of campus health services underscores the need for proactive outreach and a socioecological prevention model to enhance campus‐wide coordination.

## INTRODUCTION

Suicide remains one of the leading causes of death among young people worldwide.^1^ In Japan, it is the leading cause of death among adolescents and young adults, a pattern that is distinctive among the Group of Seven (G7) countries.^2^, ^3^ Since the onset of the COVID‐19 pandemic, a global increase in anxiety and depressive symptoms among young people has been noted.^4^, ^5^ Similarly, in Japan, a deterioration in mental health and an increase in suicide rates among youth and women have been reported since the early phase of the pandemic.^6^, ^7^, ^8^

Suicide represents the most critical consequence of mental health issues. Despite its significance, to date, relatively few studies have reported nationwide data on suicide deaths among university students. In the United States, reports exist based on student suicide data from ten universities in the Midwest (the Big Ten) over a 10‐year period in the 1990s,^9^ and another report covering the same Big Ten universities from 2009 to 2018^10^ which allowed for a decadal comparison within the same population. Other reports exist that compile a significant number of cases through public statistics or media reports,^11^ but no study covering all universities nationwide could be found.

In Japan, national universities have maintained longitudinal records on student leaves, withdrawals, and deaths—categorizing death as a reason for withdrawal—thereby providing a consistent dataset spanning more than two decades, including graduate programs.^12^, ^13^, ^14^, ^15^

Amid growing concerns about the deterioration of university students' mental health following the spread of COVID‐19, the Ministry of Education, Culture, Sports, Science and Technology (MEXT) expanded its mortality surveys—previously limited to national universities—to include all public and private universities starting in fiscal year (FY) 2020. In this study, we aggregated and analyzed data spanning the 5‐year period from FY 2020 to 2024.

## METHODS

The surveys were conducted annually between April and June from FY 2020 to 2024. MEXT issued official notices to all universities in Japan requesting their cooperation in the study. For national universities, data collection was performed by the Mental Health Committee of the Japanese National University Council of Health Administration Facilities. For public and private universities, the Japan University Health Association collected institutional responses using standardized electronic files or a dedicated web‐based form. Each organization compiled and analyzed the data received.

The survey requested the total number of enrolled students as of May 1 each year, categorized by sex and academic program. For deceased students, institutions provided demographic and academic characteristics (sex, age, enrollment status, program, and major), date of death, and cause of death classified as disease‐related, accidental, suicide (including suspected suicide), or homicide, along with situational details. In this study, “confirmed suicide” was defined as a death officially documented by the university based on police, medical, or family reports, whereas “suspected suicide” was strongly indicated by surrounding circumstances (e.g., suicide notes or recent self‐harm) without official confirmation. Deaths due to accidents, homicide, or illness were classified as non‐suicides, and cases with unknown causes were excluded from the analysis. For suicide‐related deaths, the survey further inquired about leave of absence status, the presence and diagnosis of mental disorders (with ICD‐10 codes where applicable), and prior contact with campus health services. Additional details included information on the presumed background factors for suicide (selected from 10 predefined categories; multiple answers allowed) and the estimated association with COVID‐19 (direct, indirect, none, or unknown). These data were aggregated from information accessible to the student affairs and campus health support divisions of each participating university.

Based on the collected data, we determined suicide mortality rates per 100,000 students according to sex, academic program, and institutional student population size. Differences in suicide mortality rates and methods by sex, as well as seasonality in month of death, were examined using chi‐square tests, followed by residual analyses when appropriate. Statistical analyses were performed using spss version 29 (IBM Corp., Armonk, NY, USA). The study protocol was approved by the research ethics committees of the Japan University Health Association and the Japanese National University Council of Health Administration Facilities.

## RESULTS

Across FY 2020–2024, the mean institutional response rate (based on the number of universities) was 80.3%. All 86 national universities responded in each academic year (response rate, 100%). Among public and private universities, the numbers of responding institutions (response rates) were 734 (71.2%) in FY 2020, 846 (81.0%) in FY 2021, 823 (79.8%) in FY 2022, 842 (81.3%) in FY 2023, and 827 (80.4%) in FY 2024.

As shown in Table 1, the cumulative number of enrolled students at responding universities was 13,501,304 (7,386,187 males and 6,115,117 females), representing 90.4% of the total university student population in Japan.^16^ Over this period, 1526 suicide deaths or suspected suicides (1027 males and 499 females) were identified. The mean age at the time of death by suicide was 21.8 years (SD = 3.49); both median and mode were 21 years.

**Table 1:** Suicide mortality rates per 100,000 students for FY 2020–2024.

	No. of students	No. of suicide cases	Suicide rate	*χ* ^2^	df	Adjusted residuals	*p*
Sex							
Male	73,86,187	1027	13.90	97.675	1		<0.001
Female	61,15,117	499	8.16				
Program							
Junior college	3,53,144	22	6.23	21.248	3	−2.874*	<0.001
Bachelor's	1,18,99,429	1362	11.45			1.350	
Master's	8,63,850	118	13.66			2.130	
Doctoral	3,84,881	24	6.24			−3.000*	
Population size							
−1000	8,76,332	90	10.27	1.374	3		0.712
1001−5000	36,38,757	402	11.05				
5001−10,000	34,18,335	394	11.53				
>10,000	55,66,308	640	11.50				

* Values represent adjusted standardized residuals. |r_adj| > 2.58 indicates *p* < 0.01 (two‐sided).

The male suicide mortality rate of 13.90 per 100,000 students (all mortality rates below are expressed per 100,000) was significantly higher than the female rate of 8.16 (*χ* ^2^ test, *p* < 0.001). Rates also differed by academic program (*χ* ^2^ test, *p* < 0.001). Post hoc residual analyses indicated lower rates in junior colleges (6.23) and doctoral programs (6.24) compared with undergraduate (11.45) and master's programs (13.66). Comparing suicide mortality rates by university enrollment size, no significant differences were found among the four groups (≤1000 students: 10.27; 1001–5000: 11.05; 5001–10,000: 11.53; ≥10,001: 11.50).

**Figure 1:**
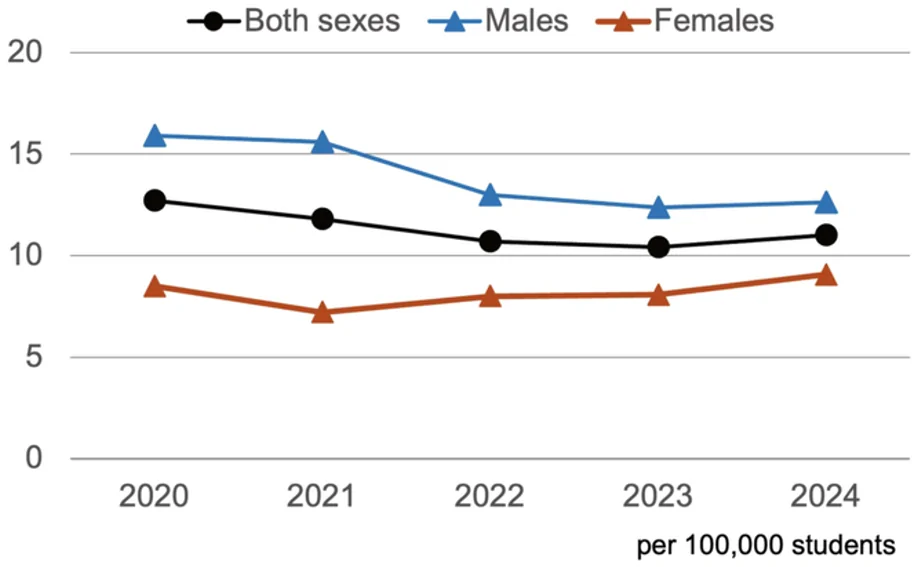
Suicide mortality rate (FY 2020–2024).

Figure 1 illustrates the annual trends in suicide mortality rates by sex over the 5‐year period (FY 2020–2024). Poisson regression analysis demonstrated a significant interaction between academic year and sex (Wald *χ* ^2^ = 6.215, IRR = 1.10, 95% CI 1.02–1.19, *p* = 0.013), indicating that temporal trends in suicide mortality differed significantly between males and females. Consequently, the difference in suicide mortality rates between males and females significantly narrowed during the study period.

Males comprised 67.4% of all suicide deaths. By age, 74.5% were 20–24 years, and 16.6% were 19 years and under. In terms of academic majors, Social Sciences and Engineering were the most prevalent at 25.5% and 22.3%, respectively, followed by Humanities at 16.6%. The most common method of suicide was hanging/suffocation (32.6%), followed by jumping (15.5%). The most frequent location was the student's own room (47.0%), whereas occurrences on university campuses were relatively rare at 2.4% (Table 2).

**Table 2:** Sociodemographic and clinical characteristis of students' suicides (1).

	No. of suicide cases	%
Sex, *n* (%)		
Males	1029	67.4
Females	497	32.6
Age		
−19	253	16.6
20−24	1136	74.5
25−29	98	6.4
30−34	15	1.0
35−39	9	0.6
40–	14	0.9
Discipline category		
Humanities	249	16.3
Social science	388	25.5
Physical science	98	6.4
Engineering	340	22.3
Agricultural science	48	3.2
Medicine and health science	161	10.6
Education	62	4.1
Art	55	3.6
Other	122	8.0
Suicide method		
Hanging/suffocation	498	32.6
Jumping	237	15.5
Gas poisoning	74	4.8
Oral poisoning	2	0.1
Overdose	30	2.0
Electrocution	9	0.6
Drowning	19	1.2
Other/unknown	657	43.1
Place of death		
Campus	36	2.4
Home	717	47.0
Car	27	1.8
Rail suicide	68	4.5
Building (excluding home and campus)	110	7.2
Outdoors (park, mountain, and sea)	162	10.6
Other	9	0.6
Unknown	397	26.0
History of temporary leave		
Yes	286	18.8
No	1238	81.2
History of repeating the same year		
Yes	285	18.7
No	1239	81.3
History of visits to campus health care facilities		
Yes	208	13.6
No	1247	81.7
Unknown	71	4.7

A history of leave of absence was identified in 18.8% of cases, and a history of repeating a year (grade retention) was found in 18.7%. Documented contact with campus health services was present in 13.6% of cases. Among the 225 cases (14.7% of the total) with available responses, after excluding 81 cases with unconfirmed diagnoses, the breakdown of the 144 cases in which a psychiatric diagnosis (ICD‐10 F‐codes) was obtained is as follows: mood disorders (F3) were the most prevalent at 56.9%, followed by neurotic, stress‐related, and somatoform disorders (22.2%). The combined proportion of developmental disorders (F8 and F9) was 9.8%, exceeding schizophrenia (8.3%). Regarding presumed suicide motives (selected from 10 categories, multiple responses allowed), academic failure was the most frequent factor (25.6%), followed by career concerns (19.8%), illness‐related worries/effects (13.3%), isolation/loneliness (12.7%), and discord in parent–child relationships (10.8%). Concerning the impact of COVID‐19, no cases were reported to have a direct association with the pandemic over the 5‐year period, and only 3.1% were identified as having an indirect association (Table 3).

**Table 3:** Sociodemographic and clinical characteristis of students' suicides (2).

	No. of suicide cases	%
Psychiatric diseases		
F1	2	1.4
F2	12	8.3
F3	82	56.9
F4	32	22.2
F5	1	0.7
F6	1	0.7
F8	7	4.9
F9	7	4.9
Diagnosis unconfirmed	81	
Presumed motives for suicide		
Academic failure	164	25.6
Career concerns	127	19.8
Job search failure	16	2.5
Personal relationships with friends/supervisors	51	8.0
Love relationship problems	36	5.6
Life hardship	11	1.7
Discord in parent–child relationship	69	10.8
Isolation/loneliness	81	12.7
Illness‐related worries/effects	85	13.3
Estimated association with COVID‐19		
Direct association	0	0.0
Indirect association	48	3.1
No association	503	33.0
Unknown	975	63.9

The monthly distribution of suicides showed significant variation (*χ* ^2^ test, *p* < 0.001). Subsequent residual analysis indicated that suicides tended to be more frequent in September and March, while being significantly fewer in August and December (Table 4). Comparing methods of suicide by sex, drowning was more prevalent among males, whereas overdose was more frequent among females (*χ* ^2^ test, *p* < 0.001; residual analysis; Table 5).

**Table 4:** Number of suicides by month.

Monthly suicides	No. of suicide cases	*χ* ^2^	df	Adjusted residuals	*p*
April	133	32.142	11	0.620	0.000724
May	131			0.434	
June	113			−1.239	
July	116			−0.960	
August	91			−3.283*	
September	151			2.292*	
October	123			−0.310	
November	123			−0.310	
December	101			−2.354*	
January	135			0.805	
February	145			1.735	
March	154			2.571*	

* Values represent adjusted standardized residuals. |r_adj| > 1.96 indicates *p* < 0.05 (two‐sided).

**Table 5:** Number of suicides by method, and by method within sex.

Method	Both sexes	Males	Females	*χ* ^2^	df	Adjusted residuals	*p*
Hanging/suffocation	498	353	145	19.004	7	2.003*	0.00817
Jumping	237	153	84			−1.027	
Gas poisoning	74	46	28			−0.992	
Oral poisoning	2	1	1			−0.526	
Overdose	30	13	17			−2.845*	
Electrocution	9	8	1			1.378	
Drowning	19	17	2			2.063*	
Other/unknown	657	438	219			−0.554	

* Values represent adjusted standardized residuals. |r_adj| > 1.96 indicates *p* < 0.05 (two‐sided).

## DISCUSSION

### Suicide mortality rates and trends

This study analyzed comprehensive nationwide data, achieving an institutional response rate of over 80% and covering more than 90% of the total university student population in Japan.^16^

Over the 5‐year study period, the suicide mortality rate for male students remained significantly higher than that for females, consistent with long‐standing epidemiological trends. When compared with official national Vital Statistics for the general population of comparable age groups (15–19, 20–24, and 25–29 years) during the study period, suicide mortality rates among university students were substantially lower. The overall suicide mortality rate was 11.30 per 100,000 students in the present study, compared with 17.65 per 100,000 in the general population (aged 15–19, 20–24, and 25–29 years); the corresponding rates were 13.90 versus 21.83 for males and 8.16 versus 13.28 for females, respectively.

In Japan, an increase in suicides among women and youth was reported in the latter half of 2020, following the onset of the pandemic.^6^, ^7^, ^17^ As shown in Figure 1, suicide mortality rates for both sexes declined from FY 2020 to 2021, after which the rate among female students showed a slight upward trend from FY 2022, whereas the male rate continued to decrease through FY 2023. Consequently, the gap between males and females continued to narrow, reaching its smallest level in FY 2024.

Among younger age groups before entering university—specifically junior high and high school students—the number of suicide deaths among females has increased consistently since 2020. According to police statistics, the number of suicide deaths among female students exceeded that among male students for the first time beginning in 2023 for junior high school students and in 2024 for high school students.^3^ Previous research has shown that among young people, females are at significantly higher risk of suicide attempts, whereas males have a significantly higher risk of suicide death.^18^ This phenomenon, commonly referred to as the “gender paradox” in suicidal behavior, varies across age groups, with the female predominance in suicide attempts peaking during mid‐adolescence.^19^ In the present study, females were significantly more likely than males to use drug overdose as a suicide method (Table 5). Although drug overdose has generally been considered less lethal than other suicide methods, the recent increase in overdoses involving readily available over‐the‐counter (OTC) medications in Japan has raised concerns that such behavior may increase the risk of progression from self‐harm or nonfatal suicide attempts to suicide. Considering the finding that a previous suicide attempt is the strongest known predictor of subsequent suicide,^20^ the narrowing sex difference in suicide mortality observed among university students may be consistent with changes in suicidal behavior that have already been reported among younger, pre‐university populations.

Regarding academic programs, while studies in the United States suggest that students aged 25 and older are at higher risk for suicide,^9^ our findings show that the mortality rate in doctoral programs—the group with the highest median age—was lower than those in undergraduate and master's programs.

Previous reports in the United States indicate that university students exhibit lower suicide mortality rates compared to age‐matched general populations.^9^, ^10^, ^21^, ^22^, ^23^ Martell and King,^10^ who compared suicide mortality rates across different periods within the same “Big Ten” universities, reported that while suicide rates in the general US population have increased, the suicide mortality rate among university students has shown a declining trend. In contrast, while nationwide census data have been limited in Japan, longitudinal mortality surveys of national university students—conducted using methods similar to the present study—have been reported for over 20 years. These indicate that while the suicide mortality rate among national university students has remained lower than that of the same‐age general population, the difference has actually narrowed in some years.^12^, ^14^

### Sociodemographic and clinical characteristics of students' suicides

Approximately three‐quarters of all suicide deaths occur among those aged 20–24, which broadly corresponds to the age range of undergraduate and graduate master's students. In the present survey, precise comparisons of suicide mortality rates across academic fields could not be conducted because data on the number of enrolled students by field were not available for the study population. However, if the number of students by field reported in Basic School Survey^16^ is used as the denominator for a tentative comparison, the risk of suicide may be relatively higher in the physical sciences and engineering fields.

Regarding the methods of suicide, excluding cases with unspecified details, hanging/suffocation and jumping accounted for the majority. It is noteworthy that sex differences were observed: hanging/suffocation and drowning were more prevalent among males, while overdose was more frequent among females. It is considered that males select suicide methods with higher fatality rates.

Approximately 19% of the students had a history of leave of absence and a similar proportion had repeated an academic year. A previous study indicated that a history of leave of absence and grade retention was associated with an increased suicide risk among national university graduate students.^12^ These academic histories may be associated with suicide risk among Japanese university students as a whole, including those at public and private universities. This observation is also consistent with the finding that academic failure was the most frequently reported presumed motive for suicide.

Only 13.6% of cases involved campus health services. This suggests that many cases resulted in suicide without receiving specialized support on campus, strongly indicating an urgent need for enhanced outreach. However, this figure does not reflect how many suicides were prevented by connecting students with high suicidal ideation to campus mental health support, so the magnitude of this proportion should be interpreted cautiously.

Information on psychiatric diagnoses (ICD‐10 F codes) was available for only 144 cases. Among these, F3 disorders accounted for more than half of the cases, and when combined with F4 disorders they comprised nearly 80%, indicating that depression and related conditions were highly prevalent. It has been suggested that most cases had a psychiatric diagnosis at the time of death.^24^ Although the number of cases with confirmed psychiatric diagnoses was limited in this study, our findings underscore the importance of depression and related conditions as the most significant psychiatric issue associated with suicide. At the same time, it may be noteworthy that developmental disorders (F8 and F9 combined) at 9.8% exceeded schizophrenia at 8.3%.

The monthly distribution of suicide deaths appeared to be associated with the Japanese academic year, which begins in April and ends in March. Regarding the period immediately after summer vacation, there have long been warnings about suicides among middle school students (ages 12–15) and high school students (ages 15–18).^25^ For university students, suicides tend to increase not only after summer break but also at the end of the academic year, when graduation and transition to employment become particularly salient. This pattern is consistent with the finding that the most frequently presumed motive for suicide was academic failure, followed by career concerns.

Regarding the impact of COVID‐19, no cases were judged to be directly related to the pandemic, and only approximately 3% were considered to have an indirect association. However, these responses were based on information available to universities, and it cannot be ruled out that methodological limitations may have influenced these findings.

### Suicide prevention on campus

Being a university student—that is, having access to various student support services on campus, including health support services—itself has been regarded as a protective factor against suicide, and this has been used to explain why suicide mortality among university students is often lower than in the same‐age general population.^10^ In the United States, restricted access to firearms on university campuses has also been discussed as an important contributing factor.^21^ While societal circumstances regarding firearms are entirely different in Japan, meaning they are not fundamentally a suicide‐related factor there, suicide prevention measures on campuses must address suicide‐related factors unique to or closely related to university students. Furthermore, with respect to protective factors against suicide, future studies are warranted to examine whether the protective effect associated with the female propensity to seek help has been attenuated in the context of university student support, including the accessibility and effectiveness of campus mental health services.

Gatekeeper Training (GKT) is the most common and widely practiced, as well as researched, method for suicide prevention in universities.^26^ In the short term, GKT has been shown to improve knowledge about suicide and self‐efficacy in responding to suicide risk.^27^, ^28^ However, it has been suggested that its effectiveness in changing help‐seeking behaviors and linking individuals to treatment is limited,^27^ and evidence for improvements in long‐term knowledge and behavioral outcomes related to suicide has not been consistently demonstrated.^28^

In Japan, there are few studies examining the current state of suicide prevention measures in universities. The authors previously conducted a situation survey specifically for national universities.^29^ Alongside assessing the current situation, development and pilot testing of effective programs feasible for implementation at universities are underway.^30^ Future studies should evaluate the effectiveness of such programs, and their broader implementation across universities would be desirable.

Furthermore, suicide prevention should not be limited to approaches targeting individuals or specific groups within the campus community. Rather, it should be strengthened based on a socioecological model^31^ that encompasses campus‐wide campaigns, screening programs, institutional policies, and organizational structures for suicide prevention, including planning and administrative coordination at the university level.

### Limitations

This survey was based on mortality information accessible to university administrations. Consequently, it is possible that some suicide cases were categorized as “unknown” or “unspecified” in the survey responses. It is not possible to accurately assess the extent to which these “dark figures” (unreported cases) may have influenced the overall findings of this aggregation.

## CONCLUSION

This 5‐year nationwide study provides the most comprehensive epidemiological assessment of suicide among university students in Japan, covering over 90% of the student population. Our findings highlight that while male suicide rates remain significantly higher, the sex difference is narrowing in alignment with trends observed in younger, pre‐university populations. The data identify critical risk factors, particularly academic failure and career concerns, which correspond to seasonal peaks in September and March—the key transitional periods of the Japanese academic year. Despite these risks and mental health issues—predominantly depression—identified as significant, the low utilization rate of on‐campus mental health services underscores the critical need for more proactive outreach. Effective suicide prevention should transcend individual‐level interventions and adopt a socioecological model that enhances coordination across the campus community.

## AUTHOR CONTRIBUTIONS

**Katsuhiro Yasumi**: Writing—original draft; writing—review and editing; methodology; validation; investigation; formal analysis; data curation; project administration. **Takehiro Ibaraki**: Writing—review and editing; methodology; validation; investigation; formal analysis; data curation. **Yasuko Fuse‐Nagase**: Writing—review and editing; methodology; validation; investigation; formal analysis; data curation. **Toshiyuki Marutani**: Writing—review and editing; methodology; validation; investigation; formal analysis; data curation. **Hirokazu Tachikawa**: Methodology; validation; investigation; supervision. **Asumi Takahashi**: Methodology; validation; investigation; supervision. **Terumi Ishii**: Methodology; validation; investigation; supervision. **Toshinari Odawara**: Methodology; validation; investigation; supervision. **Chiaki Kawanishi**: Methodology; validation; investigation; supervision.

## CONFLICT OF INTEREST STATEMENT

The authors declare no conflicts of interest.

## ETHICS APPROVAL STATEMENT

The study protocol was approved by the research ethics committees of the Japan University Health Association and the Japanese National University Council of Health Administration Facilities.

## PATIENT CONSENT STATEMENT

N/A.

## CLINICAL TRIAL REGISTRATION

N/A.

## Data Availability

Data are available from the authors upon reasonable request and with the permission of the relevant institutions.
